# The Effect of Positive Intervention Dosing Frequency: Fixed Intervals May Decrease More Depression than Flexible Ones

**DOI:** 10.3390/ijerph19159227

**Published:** 2022-07-28

**Authors:** Sen-Chi Yu, Chun-Wei Chang

**Affiliations:** Department of Counseling and Applied Psychology, National Taichung University of Education, Taichung 40306, Taiwan; rhine@gm.ntcu.edu.tw

**Keywords:** positive psychology, positive interventions, depression, intervention dosing

## Abstract

Positive interventions (PIs) that are based on the theory of positive psychology have proven to be effective in improving well-being and alleviating depression. However, little research has explored the effect of dosing intervals on experimental effects. As such, this study designed strength-based PIs using cognitive reframing theory and compared flexible and fixed dosing intervals to find out which one could more effectively reduce depression with equal total amounts of dosing. The 8-item Center for Epidemiological Studies Depression Scale (8-item CES-D) and the Positive reframing scale (PRS) were adopted as research instruments. A total of 193 Taiwanese college students were recruited as the research sample and they were randomly assigned to experimental Group A (fixed dosing intervals), experimental Group B (flexible dosing intervals), and the Control Group. The research participants received 17-day interventions with follow-up tests administered in the seventh week of the experiment. Ultimately, 157 participants completed the experiment. According to the ANCOVA results, participants in experimental Group A showed significantly lower degrees of depression than those in the Control Group in both post-test and follow-up stages and displayed greater effect size in the follow-up stage than in the post-test stage. The results indicated that the design of fixed dosing intervals enabled the participants to effectively integrate reflections on reframing learned during PIs into their life. On the contrary, participants in experimental Group B exhibited no significant difference in the degree of depression from those in the Control Group during either the post-test or follow-up stage and manifested poorer effects in the follow-up stage than in the post-test stage. These results demonstrated that fixed dosing intervals achieved better effects than flexible dosing intervals. Participants receiving fixed dosing intervals could more effectively execute cognitive reframing and showed longer-lasting experimental effects, whereas participants using the design of flexible dosing intervals were more prone to forget to implement PIs and attain less positive effects as a result.

## 1. Introduction

Positive psychology is the scientific study of life most worth living, well-being, and human flourishing, and approaches to optimal functioning [1,2]. Research on positive psychology-based positive interventions (PIs) has demonstrated that such PIs may help people properly exert their character strengths in their lives, and accordingly generate positive effects on different aspects of their lives [3]. PIs have proven to be effective in enhancing people’s positive emotions [1,2,4,5,6] and mitigating depression [1,2,6,7,8]. Despite the proven effectiveness of PIs, the author of this study deems PIs integrated with reframing technologies and dosing intervals for interventions to be two research topics worth investigating.

Cognitive adjustment is a major factor in behavior change. Cognition-oriented reframing, including cognitive restructuring and cognitive reappraisal, enables individuals to positively reshape their existing experiences in a more adaptive and effective manner [9,10]. Reframing may facilitate adjustments on the cognitive level and render an understanding of problems or experiences in a more positive and out-of-the-box way, thereby increasing people’s confidence in themselves and prompting them to face challenges with an open attitude. Changes in the cognitive, emotional, and behavioral domains require repeated practice. Shapira et al. [11,12,13,14] conducted a series of studies with older adults and validated that behavioral and cognitive techniques can be learned and practiced via an online program. Research shows that online training in behavioral and cognitive techniques could reduce loneliness, depression [11,14], mental stress and promote well-being [12]. In their empirical research, Stoeber and Janssen [15] discovered that reframing can effectively aid apprehensive-type perfectionists in recognizing their personal strengths, help them better address frustrations and pressures in their lives, and enhance their life satisfaction levels. Padesky and Mooney [16] indicated that when counselors engage in dialogue with clients to reframe their knowledge of personal character strengths and develop new actions in response, they may help their clients build resilience to confront challenges. Nevertheless, relatively little existing research on PIs has explored the topic by integrating cognitive technologies into interventions [1,5,7,17].

Behavioral intervention dosing may be characterized by duration, frequency, and amount [18]. Intervention dosing is a crucial factor influencing experiment results. An appropriate dosing design will further regulate people’s motivation and perception when intervention programs are being implemented in their daily life, thereby enabling PIs to achieve maximum effects [19]. In terms of frequency, Lyubomirsky et al. [20] suggested that repetitive and excessive PIs will cause a sense of weariness in individuals and diminish the effectiveness of interventions as a result. Lower-frequency intervention doses can sometimes more effectively maintain a sense of freshness towards intervention programs in individuals while further inducing them to develop motivation for continuous adoption of a given strategy in the future. Regarding the determination of frequency, some studies performed activities using a fixed frequency. For example, previous research required participants to carry out intervention activities on a daily basis over a one-week intervention period, and research results revealed that interventions can promote their mental well-being and reduce depression [1,5,6,7,16]. However, other studies have discovered that if the intervention period is extended to more than one week, during which time participants are allowed to freely determine the frequency of execution, interventions are equally effective in promoting well-being despite variations in the frequency of program execution among participants [1,4,21]. Further examination revealed that the longer the intervention time, the more flexibility the participant had to make in performing the activity. This makes it possible to avoid overuse of the activity due to the prolonged intervention time, which would cause the participant to feel satiation in the perception and stop benefiting from it [22]. Lyubomirsky, Sheldon, and Schkade [20] emphasized that behavior changes, characterized by sporadicity and diversity, can help us break through hedonic adaptation, and therefore, behavior changes generated by personal intentions can deliver a longer-lasting sense of well-being than environmental changes. The concentration of intervention doses, exerting the strength of benevolence five times in a day achieves higher concentration than doing so five times in a week can help individuals avoid the outcome in which positive perceptions are lessened as a result of habituation and reduced freshness induced by constant activity execution during the intervention period [20]. Dosing intervals are a crucial factor influencing experimental effects. Whether fixed dosing intervals preset by researchers or participant-customized dosing intervals can produce better effects remains to be investigated.

Based on the above discussions, this study designed PIs using reframing technologies as a foundation, and compared flexible and fixed dosing intervals with a controlled total amount of dosing to find out which one can more effectively reduce depression.

It was hypothesized that: (1) the flexible interval group and Control Group would display significant differences in the post-tests and follow-up stages. (2) the fixed interval group and Control Group would display significant differences in the post-tests and follow-up stages. (3) the flexible and fixed dosing interval will displayed significant differences in the post-tests, and follow-up stages.

## 2. Methods

### 2.1. Participants and Procedure

The initial sample consisted 199 college students. The inclusion criteria were: college students age 20 to 24; with internet access. The exclusion criteria were: age below 20 years; individuals with pre-existing mental illness and under treatment. After applying the exclusion criteria, 6 students aged below 20 were excluded and 193 eligible students (62 men and 131 women, median age 21). were recruited to participate in this study and randomly assigned them to experimental Group A, experimental Group B, and the Control Group.

**Figure 1:**
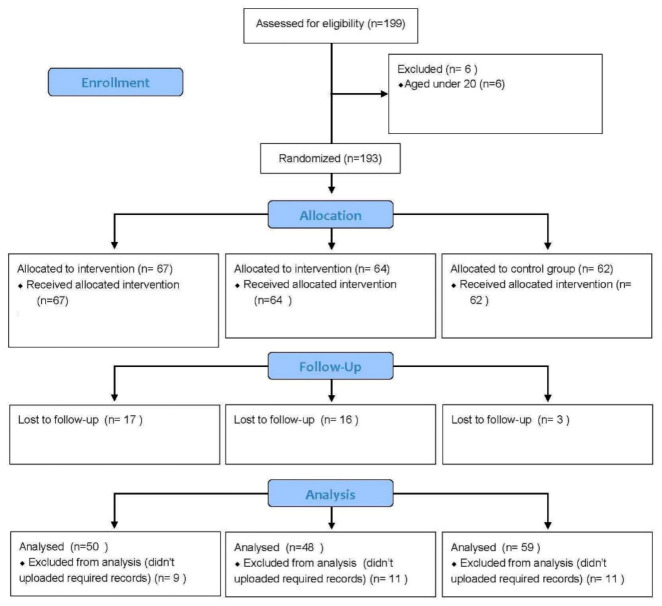
Flow chart of this study.

A total of 157 people completed the experiment, with an overall attrition rate of 18.65%. Participants who didn’t uploaded required records of the intervention were excluded from the analysis. The numbers of participants in experimental Group A, experimental Group B, and the Control Group completing the pre-test stage were 67, 64, and 62, respectively, and the numbers of those completing the entire experiment were 50, 48, and 59, respectively, reaching the attrition rates of 25.37%, 25%, and 4.83%, respectively. Ethics approval for the study was provided by The Institutional Review Board of National Chung Cheng University (Ethical Application Ref: CCUREC107020603). The flowchart (Figure 1) illustrates the number of participants in the different groups and during the study stages.

After obtaining consent from the instructors, the researcher explained to the participants in each class the strengths examined in this study, the benefits of participating in the experiment, experimentation ethics, data confidentiality, and other matters. Next, the participants were requested to sign a consent form, while also being promised a gift with a market value of approximately NTD100 upon completing the entire activity as required by the researcher. The participants were randomly assigned to three groups. The intervention period lasted for 17 days, during which time the experimental groups performed designated PI activities, whereas the Control Group completed news-sharing assignments.

Our positive intervention was adapted from “The Three Good Things in Life” and “You at Your Best” [1]. The researcher posted introductory information regarding reframing on a website. Following the steps of PI activities, the website guided the participants to begin the process of reframing and conduct the 17-day self-reflections and strength explorations. The experimental procedure is detailed as follows:

(1) In the first week of the experiment, the participants were required to observe one particular experience of positive emotions in their lives on a daily basis. A total of seven experiences should be recorded by taking photos and answering the reflection questions on the research website provided by the researcher. This procedure used “attention technique”, the prerequisite for cognitive learning, to discover and pay attention to positive events in life.
(2) From the eighth day through the tenth day of the experiment, the participants were required to read the content of the “Strengths and Virtues” page on the website. In case of any doubt, the participants could ask the researcher questions through instant messaging. This part is cognitive-level learning through reading and provides relevant knowledge and cognitive schema for the next experimental procedure.
(3) From the eleventh day to the seventeenth day of the experiment, the participants were required to reflect on their previously recorded positive emotion experiences from a character strength-based cognitive perspective as per the chronological order of the records kept in the first activity “Good Things in Life.” This procedure is to enhance positive emotion through cognitive reframing.
  Participants in Group A (fixed dosing interval) performed reflections at a frequency of one record per day, thus completing a total of seven reflection records. Participants in Group B (flexible dosing interval) generally followed the same experimental procedure, except that they were allowed to determine their preferred frequency at which “Good Things in Life” reflection records were kept, while also completing a total of seven reflection records. Participants in the Control Group completed the sharing of seven news stories at this stage and recorded their sharing on the website established by the researcher.
(4) A follow-up test was administered in the seventh week of the experiment, and participants’ feedback and reflections on the experiment process were collected.

### 2.2. Instruments

#### 2.2.1. 8-item Center for Epidemiological Studies Depression Scale (8-item CES-D)

Center for Epidemiological Studies Depression Scale, CES-D is a four-point, 20-item scale that assesses depression symptoms [23]. CES-D is one of the most widely used depression scales in the world and has been translated and verified for reliability and validity in a number of languages [7]. This study applied the Chinese version 8-item CES-D developed by Yu et al. [24]. The 2-week and 4-week test–retest reliability were 0.917 and 0.825 [24]. The Cronbach alpha was 874 in our study, indicating good reliability.

#### 2.2.2. Positive Reframing Scale (PRS)

To check whether participants used reframing techniques during PIs, we used the positive reframing scale, a subscale of the Brief COPE Scale [25], at post-test and follow-up test stages. PRS is a two-item, four-point scale. The items in PRS are *“ I’ve been trying to see things in a different light, to make it seem more positive.”* and *“ I’ve been looking for something good in things that happening.”*

### 2.3. Statistical Analyses

To determine whether there are significant differences between groups on depression scores, we performed one-way ANCOVA (Analysis of covariance) on collected data using SPSS 27.0 statistical software. By using pre-test scores as the covariate to control for pre-existing differences on the dependent variable, The ANCOVA looks for differences in adjusted means (i.e., adjusted for the covariate/pre-test scores). In this study, we used pre-test CESD scores as covariate and post- and follow-up CES-D scores as dependent variables separately to discuss the intervention effectiveness of the two stages.

## 3. Results

### 3.1. Preliminary Analyses of Participants’ Reframing

Participants having completed all PI activities and uploaded all required records totaled 50 and 48 for Group A and Group B, respectively. According to the items checking the use of reframing skills in the post-test survey on participants of the two experimental groups, 61.2% reported that they would sometimes “try to look at a problem from a different perspective to make the problem more positive” during the 17-day intervention program, and 35.7% expressed they would frequently do so. In addition, 44.9% of the participants stated that they would sometimes “find out positive meanings from what has happened” during the 17-day intervention program, and another 44.9% indicated they would frequently do so.

In the follow-up test survey, 52% of the participants in the experimental groups reported that they would sometimes “try to look at a problem from a different perspective to make the problem more positive” in the two weeks following the intervention program, and 41.8% stated that they would do so frequently. In addition, 57.1% of the participants in the experimental groups expressed that they would sometimes “find out positive meanings from what has happened” in the two weeks following the intervention program, and 38.8% noted that they frequently do so.

The above statistics reveal that the majority of the participants in the experimental groups have learned reframing skills by following the guidance of the intervention program and relevant steps and further applied the skills to practice.

### 3.2. Statistical Analysis of Intervention Effectiveness

**Figure 2:**
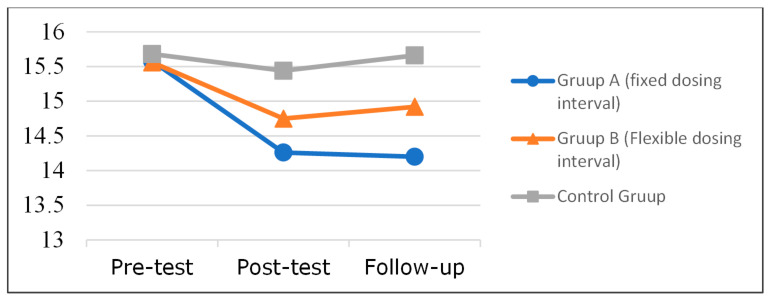
CES-D means by group at time 1 (pre), time 2 (post), and time 3 (follow-up).

The results of mean and SD at the three-time periods for CES-D scores are shown in Table 1. Graphically, according to Figure 2, Group A displayed reduced depression in the post-tests, and this reduction continued through to the follow-up. Group B displayed reduced depression in the post-tests, with slightly higher depression scores observed in the follow-up stage than in the post-test stage. The Control Group exhibited little variation in depression scores.

The results of post-test ANCOVA are shown in Table 2 and Table 3. Concerning the homogeneity of variance between groups, the Levene test results found homogeneity hold for both the post-test (*p*> 0.05) and follow-up stage (*p* > 0.05). The test of homogeneity of within-group regression coefficient found homogeneity held for both post-test (*p* > 0.05) and follow-up stage (*p* > 0.05).

As for the post-test stage, omnibus ANCOVA results revealed no significant difference between the three groups (F(2,153) = 2.414, *p* > 0.05). However, the post hoc tests (as shown in Table 3) found that the means of Group A was lower than Group C significantly (*p* < 0.05). The effect size (Cohen’s d) was 0.31, reaching a small to medium effect size. For Experimental Group B, no significant differences were found between Group B and the Control Group (*p* > 0.05).

For the follow-up stage (as shown in Table 4 and Table 5), omnibus ANCOVA results revealed significant differences between the three groups (F(2,153) = 4.307, *p* < 0.05). The omnibus effect size (Partial eta squared) = 0.53. The post hoc tests found that the means of Group A was lower than Group C significantly (*p* < 0.05). The effect size (Cohen′s d) was =0.40, reaching a small to medium effect size. However, no significant differences were found between Group B and C (*p* > 0.05).

To sum up, Group A and the Control Group displayed significant differences in the post-tests, and continued through to the follow-up stages. However, no significant differences were found between Group B and the Control Group in both stages.

## 4. Discussion

In this study, college students in Group A displayed reduced levels of depression in both post-test and follow-up stages, but those in Group B did not exhibit reduced levels of depression in either the post-test stage or follow-up stage.

The results of Group A derived in this study accord with those of most PI-related studies, indicating that fixed dosing intervals can expand one’s character strengths and effectively reduce depression [1,5,6,8]. Combining feedback from the participants and related research results [17,26], this study inferred that interventions with fixed dosing intervals were able to remind the participants to change their habits, take some time each day to review and reflect on their personal experience and adjust their way of thinking by focusing on their character strengths. These actions enabled the participants to discover and recognize their personal qualities and abilities, while further establishing their own values, such as clarifying how their character strengths contributed to their personal interests, daily life or goals and identifying effective personalized strategies to lead better lives. Some participants also reported that they would apply the reflection approaches learned from the program to confront difficulties, which enabled them to exert their character strengths to resolve the difficulties.

The aforementioned results are consistent with those obtained by other researchers, indicating that when interventions with fixed dosing intervals increase positive self-perceptions, they effectively alleviate depression [17]. In addition, when their cognition is broadened, the participants may be prompted to establish their personal resources [26]. Furthermore, the researcher reckons that the fixed dosing interval design may help participants to gradually incorporate new perceptions into their self-concept system [27], which would have an amplifying effect on reducing depression.

However, experimental Group B achieved no significant difference from the Control Group in the scores of both post-test and follow-up test, and displayed poorer effects in the follow-up stage than in the post-test stage. The researcher argues that the flexible dosing interval design could not prevent habituation or hedonic adaptation [20]. In addition, due to the nature of the flexible dosing interval design, participants might easily forget to execute PIs or perform intervention activities. They might also postpone doing so when interfered with by other to-do items with greater urgency, which made newly perceived content less likely to be retained in their perception. Moreover, because the program did not require participants to take corresponding substantive actions in response to cognitive changes, this study assumes that interventions adopting the flexible dosing interval design were less likely to induce changes in the depression levels of the participants.

In terms of sustained effects, the researcher speculates that the difference between the two experimental groups may have resulted from the fact that variations in the frequency of intervention had an influence on the level of cognitive change in the participants. In addition to the understandings recorded by the participants regarding their character strengths, expanding perception levels through consistent practice in the context of life experience might help change the participants’ other implicit cognitions in relation to their selves while further sustaining their changes even after the intervention. On the other hand, for participants in the group adopting a flexible dosing interval design, their knowledge of personal character strengths was not underscored during the intervention period. As such, returning to their regular life with a lesser extent of cognitive change, they were less likely to observe changes in depression levels.

## 5. Limitation

Several limitations to the present study should be noted. First, the PI adopted in this study was aimed at cognitive reframing. Cognitive change may require a longer time with fixed dosing intervals to achieve better experimental results. Whether the results of this study are applicable to PIs that place less emphasis on cognitive change remains to be verified.

In addition, the dependent variable in this study (depression) tilted toward negative emotions. According to related research on positive and negative emotions, positive and negative emotions do not offset each other equally [28]. Therefore, whether the results of this study can be generalized to positive emotions (e.g., happiness and well-being) is worthy of further investigation.

Few existing studies have discussed cultural differences in PIs [1]. The sample of this study (college students in Taiwan) belonged to a collectivist culture. Whether individuals in a collectivist culture displayed higher levels of obedience, more strictly adhered to intervention intervals preset by the researcher, and thus achieved better effects in the intervention is also worthy of in-depth discussion [1]. Moreover, the potential self-selection bias should be considered.

## 6. Conclusions

This study explored whether flexible dosing intervals or fixed ones could more effectively reduce depression. A total of 193 college students in Taiwan were recruited and randomly assigned to experimental Group A (fixed dosing), experimental Group B (flexible dosing), and the Control Group through the randomized controlled trial (RCT) approach. The participants received 17-day interventions. According to the ANCOVA results, the group adopting fixed dosing obtained significantly lower scores than the Control Group in 8-item CES-D post-test and follow-up tests, and displayed greater effect size in the follow-up stage than in the post-test stage. The results demonstrate that fixed dosing PIs enabled the participants to effectively apply reflection approaches learned during PIs to confront challenges and further integrate the approaches into their lives. On the contrary, experimental Group B exhibited no significant difference from the Control Group in either post-test scores or follow-up scores, and manifested poorer effects in the follow-up stage than in the post-test stage. These results demonstrated that fixed dosing intervals achieved better effects than flexible dosing intervals. Participants receiving fixed dosing intervals could more effectively execute cognitive reframing and showed longer-lasting experimental effects, whereas participants using the design of flexible dosing intervals were more prone to forget to implement PIs and unable to prevent habituation.

## Figures and Tables

**Table 1:** Mean and SD of the three conditions at the three-time periods for CES-D scores.

	Pre-Test		Post-Test			Follow-up Test		
	Mean	SD	Mean	SD	Adj. Mean	Mean	SD	Adj. Mean
Group A	15.58	3.208	14.26	3.250	14.28	14.20	3.591	14.23
Group B	15.56	3.524	14.75	3.118	14.79	14.92	3.695	14.96
Control	15.68	3.165	15.44	4.280	15.39	15.66	3.637	15.61

Note: Adj. Group A = fixed dosing interval group; Group B = flexible dosing interval group; Control = Control Group; Adj. Mean = the covariate-adjusted mean.

**Table 2:** Summary of ANCOVA Results of Post-test.

Source	SS	df	MS	F	*p*	Partial $\eta^{2}$
Intercept	53.681	1	53.681	7.766	0.006 **	0.048
Pre-test CESD	979.567	1	979.567	141.712	0.000 ***	0.481
group	33.378	2	16.689	2.414	0.093	0.031
Error	1057.595	153	6.912			
Total	36,714.000	157				

Note. ** *p* < 0.01. *** *p* <0.001.

**Table 3:** Pairwise comparisons between groups of the post-test stage.

(I) Group	(J) Group	Mean Difference (I-J)	Std. Error	*p*-Value
A	B	−0.503	0.531	0.345
A	C	−1.106	0.505	0.030 *
B	C	−0.602	0.511	0.241

Note. * *p* < 0.05.

**Table 4:** Summary of ANCOVA Results of Follow-up—test.

Source	SS	df	MS	F	*p*	$\mathrm{Partial}\;{}^{2}$
Intercept	29.329	1	29.329	4.898	0.028 *	0.031
Pre-test CESD	1124.666	1	1124.666	187.808	0.000 ***	0.551
group	51.586	2	25.793	4.307	0.015 *	0.053
Error	916.221	153	5.988			
Total	37,274.000	157				

Note. * *p* < 0.05, *** *p* < 0.001.

**Table 5:** Pairwise comparisons between groups of follow-up stage.

(I) Group	(J) Group	Mean Difference (I-J)	Std. Error	*p*-Value
A	B	−0.731	0.494	0.141
A	C	−1.381	0.470	0.004 *
B	C	0.731	0.494	0.141

Note. * *p* < 0.05.

## Data Availability

The data presented in this study are available on request from the first author.
